# Effect of ambrisentan in patients with systemic sclerosis and mild pulmonary arterial hypertension: long-term follow-up data from EDITA study

**DOI:** 10.1186/s13075-024-03363-0

**Published:** 2024-07-18

**Authors:** Panagiota Xanthouli, Paul Uesbeck, Hanns-Martin Lorenz, Norbert Blank, Christina A. Eichstaedt, Satenik Harutyunova, Benjamin Egenlauf, Jerry G. Coghlan, Christopher P. Denton, Ekkehard Grünig, Nicola Benjamin

**Affiliations:** 1 Centre for Pulmonary Hypertension, Thoraxklinik Heidelberg GmbH at Heidelberg University Hospital, Röntgenstraße 1, 69126 Heidelberg, Germany; 2 Translational Lung Research Centre Heidelberg (TLRC), German Centre for Lung Research (DZL), Heidelberg, Germany; 3 Department of Pneumology and Critical Care Medicine, Thoraxklinik Heidelberg gGmbH at Heidelberg University Hospital, Heidelberg, Germany; 4 Department of Internal Medicine V: Hematology, Oncology and Rheumatology, University Hospital Heidelberg, Heidelberg, Germany; 5 Cardiology Department, Royal Free Hospital, London, UK; 6 Centre of Rheumatology, Royal Free Hospital, London, UK

**Keywords:** Early treatment, Ambrisentan, Systemic sclerosis, Pulmonary vascular disease

## Abstract

**Background:**

In the EDITA trial, patients with systemic sclerosis (SSc) and mild pulmonary vascular disease (PVD) treated with ambrisentan had a significant decline of pulmonary vascular resistance (PVR) but not of mean pulmonary arterial pressure (mPAP) vs. placebo after six months. The EDITA-ON study aimed to assess long-term effects of open label therapy with ambrisentan vs. no pulmonary arterial hypertension (PAH) therapy.

**Methods:**

Patients who participated in the EDITA study and received regular follow-up were included in EDITA-ON. Clinical, echocardiographic, laboratory, exercise and hemodynamic parameters during follow-up were analysed. The primary endpoint was to assess whether continued treatment with ambrisentan vs. no treatment prevented the development of PAH according to the new definition.

**Results:**

Of 38 SSc patients included in the EDITA study four were lost to follow-up. Of the 34 remaining patients (age 55 ± 11 years, 82.1% female subjects), 19 received ambrisentan after termination of the blinded phase, 15 received no PAH medication. The mean follow-up time was 2.59 ± 1.47 years, during which 29 patients underwent right heart catheterization. There was a significant improvement of mPAP in catheterised patients receiving ambrisentan vs. no PAH treatment (-1.53 ± 2.53 vs. 1.91 ± 2.98 mmHg, *p* = 0.003). In patients without PAH treatment 6/12 patients had PAH vs. 1/17 of patients receiving ambrisentan (*p* < 0.0001).

**Conclusion:**

In SSc patients with early PVD, the development of PAH and/or deterioration was less frequent among patients receiving ambrisentan, indicating that early treatment and close follow-up could be beneficial in this high-risk group. Future trials in this field are needed to confirm these results.

## Introduction

Systemic sclerosis (SSc) is a rare and complex rheumatic disease characterised by fibrosis, vasculopathy and autoimmunity [1]. Patients with SSc are at high risk for development of pulmonary hypertension (PH) at any stage of the disease [2]. Screening programs such as the DETECT algorithm are recommended and validated for the early detection of PH in this risk population [3]. Guidelines support yearly PH screening of patients with SSc especially in case of diffusion capacity disorder [4].

Early detection of pulmonary vascular disease (PVD), before the progress to a severe impairment of hemodynamics, leads to improvement of survival in this population [5]. Although survival of patients with pulmonary arterial hypertension (PAH) has overall improved in the last decades, the mortality rate of SSc-associated PAH patients still remains high [6–8]. The definition of PAH in the 2022 ESC/ERS guidelines aims to identify patients at even earlier stages of the disease [4]. The treatment indication of PAH targeted therapy in early PVD stages remains unclear, though.

The EDITA trial aimed to investigate the effect of ambrisentan, an endothelin receptor antagonist approved for the treatment of PAH, on hemodynamics, in particular on mean pulmonary arterial pressure (mPAP) in SSc patients with mild PVD [9]. Patients with mildly elevated mPAP and/or exercise PH treated with ambrisentan in the EDITA trial had a significant decline of pulmonary vascular resistance (PVR) but not of mPAP vs. placebo after 6 months. The treatment was overall well tolerated [9].

After study termination, a number of patients received open label ambrisentan to prevent the development of PAH and patients were followed according to PH guidelines valid during the time of assessment. This is the first, long-term, follow-up evaluation of patients that participated in the EDITA study (EDITA-ON) aiming to assess the efficacy and effects of treatment with ambrisentan vs. no PAH treatment on hemodynamics, exercise capacity, echocardiographic and laboratory parameters, as well as the prevention of development of severe PVD.

## Methods

### Study design and participants

In this single-centre, retrospective cohort study, diagnosis of adult SSc was confirmed by experienced rheumatologists (HML, NoB, PX) according to the standard criteria of the American College of Rheumatology/European League against Rheumatism [10]. Patients that participated previously in the EDITA study and continued regular screening and follow-up visits in the centre for PH in the Thoraxklinik at Heidelberg University Hospital in Germany were included in the EDITA-ON study. The EDITA study was a single center, investigator-initiated trial using a prospective, randomized, double-blind, parallel group, placebo-controlled, phase IIA clinical study design [9]. Patients were randomized 1:1 to either ambrisentan or placebo by simple randomization. The inclusion/exclusion criteria of the former EDITA trial were previously reported [9]. SSc patients were referred to our center for the purpose of PH screening which was performed according to a modified DETECT algorithm and were enrolled into the EDITA study from December 2014 until April 2017 if they had either (1) resting mPAP 21–24 mmHg, PAWP < 15 mmHg, transpulmonary gradient (TPG = mPAP - PAWP) > 11 mmHg, and/or (2) exercise induced elevated mPAP-values > 30 mmHg, PAWP < 18 mmHg, TPG > 15 mmHg which occurred at low workloads (cardiac output (CO) < 10 l/min) without significant left heart or severe interstitial lung disease. Inclusion of patients was based on pulmonary arterial pressures and not on PVR, as this criterion was not yet implemented during the EDITA study. To exclude PH due to other diseases left heart disease was assessed/excluded by clinical examination, electrocardiogram (ECG), echocardiography, stress echocardiography, stress ECG and laboratory testing of the N-terminal fraction of the pro brain natriuretic peptide and troponin T. Patients suspected of having coronary artery disease or any other left heart disease, including all with elevated wedge pressures were referred for left heart catheterization. Lung disease was assessed/excluded by lung function tests, chest X-ray and if clinically indicated by high resolution computed tomography (HRCT). Lung involvement of SSc was considered significant when forced vital capacity (FVC) < 60%, or HRCT showed severe fibrosis, or when FVC was 60-70% and HRCT showed moderate-severe fibrosis or was „not available“. Manifest PH/PAH was diagnosed according to the ESC/ERS-guidelines [9, 11]. At the end of the EDITA trial, patients of the ambrisentan and the placebo group were either offered to continue or to newly start with the drug which was then prescribed for clinical reasons to prevent worsening of the early PVD. Physicians and patients decided in an open discussion if ambrisentan treatment should be performed or not. Treatment decisions were not part of the EDITA-ON trial which aimed to document the effects of this treatment by regular follow-up visits between 08/2015 and 03/2023, which were done according to routine and guideline recommendations.

The Ethics Committee of the Medical Faculty of Heidelberg University Hospital raised no concerns to the execution of the study (internal number S-377/2023). The study adhered to the latest version of the Declaration of Helsinki. All data were pseudonymized. A written informed consent was provided from all participating patients after detailed information and discussion with the study physician.

### Study parameters

After the end of the EDITA study [9] patients performed regular follow-up visits according to clinical routine (with visit intervals between 3 and 12 months according to patient risk, signs and symptoms) including medical history, detailed clinical examinations, documentation of signs of clinical worsening, echocardiography, pulmonary function tests, diffusion capacity measurements for carbon monoxide (DLCO), 6-minute walking distance (6MWD), evaluation of World Health organization (WHO) functional class (FC) and laboratory parameters such as blood count, renal and liver function parameters, NTproBNP and uric acid. Patients with signs of progression of PVD underwent a hemodynamic re-evaluation with right heart catheterization (RHC).

### Efficacy variables

The aim of this study was to evaluate clinical routine data on the effect of ambrisentan in preventing deterioration of PVD in a high-risk population with SSc. In addition, the impact of selective ambrisentan therapy on routine clinical parameters was assessed to support the primary endpoint. Particularly, the deterioration of mPAP and the change in pressure values were examined and compared between the groups by use of categories including the new hemodynamic definition [4] with exercise-induced PH, manifest PH and normal pressures.

### Statistical methods

Data are described as mean ± standard deviation or n and respective percentage for frequency data. Change of mPAP was compared between groups with an analysis of variance with baseline values as covariates and an analysis of variance for change in mPAP in consideration of treatment in both EDITA and EDITA-ON. Comparison of categorical data was performed with chi-square tests. Clinical data at baseline and changes in clinical parameters between the end of the EDITA trial and last follow-up were compared between groups with student’s t-test or nonparametric testing as appropriate (Wilcoxon-Mann-Whitney or chi square test).

P-values < 0.05 were considered statistically significant. Due to the exploratory nature of the study, no adjustment for multiple testing and no imputation of missing values was performed. Data was analysed with IBM SPSS V27.0 (SPSS Statistics V27, IBM Corporation, Somers, New York).

## Results

### Baseline characteristics (Table 1; Fig. 1)

**Table 1:**
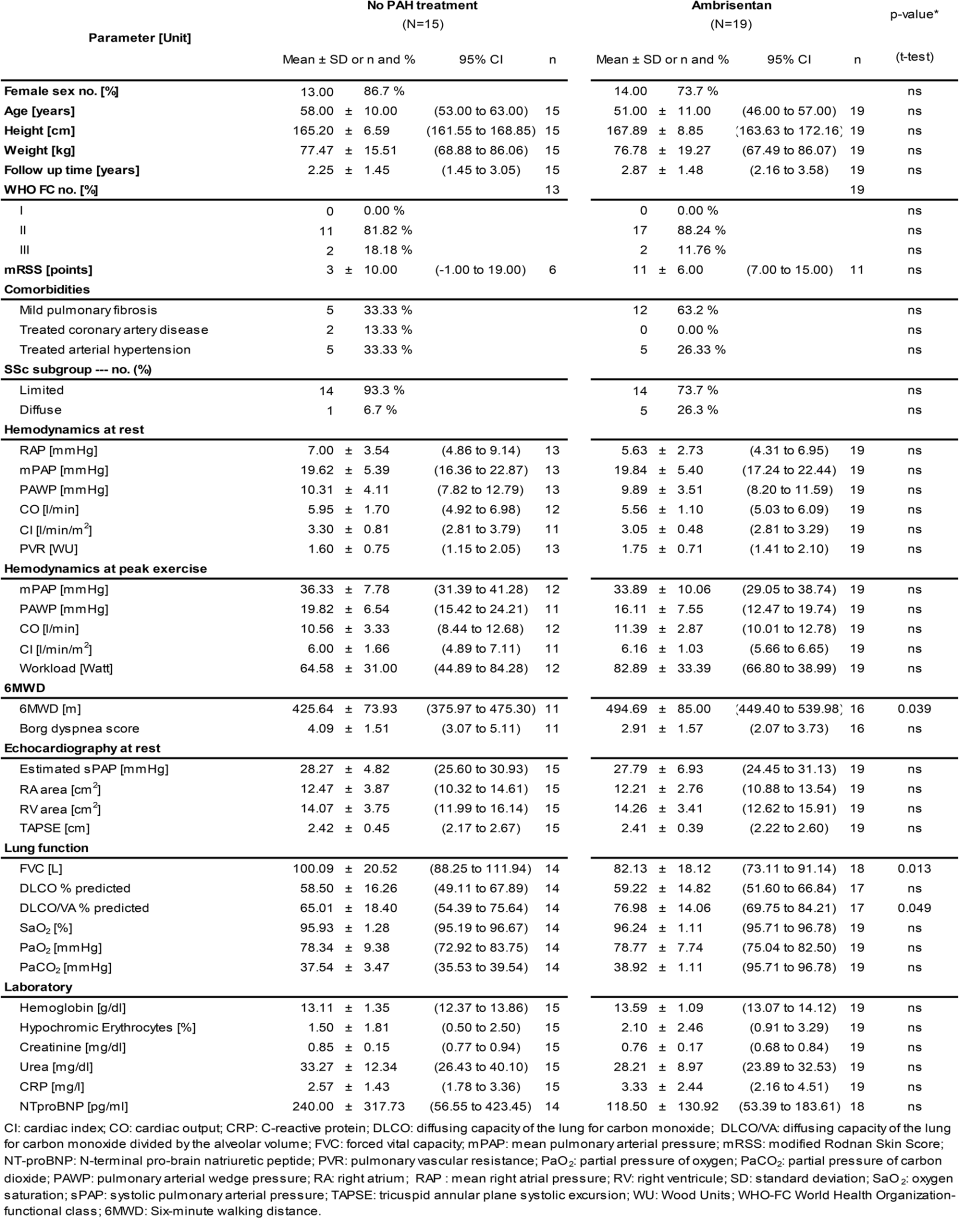
Baseline characteristics of the study cohort

**Fig. 1:**
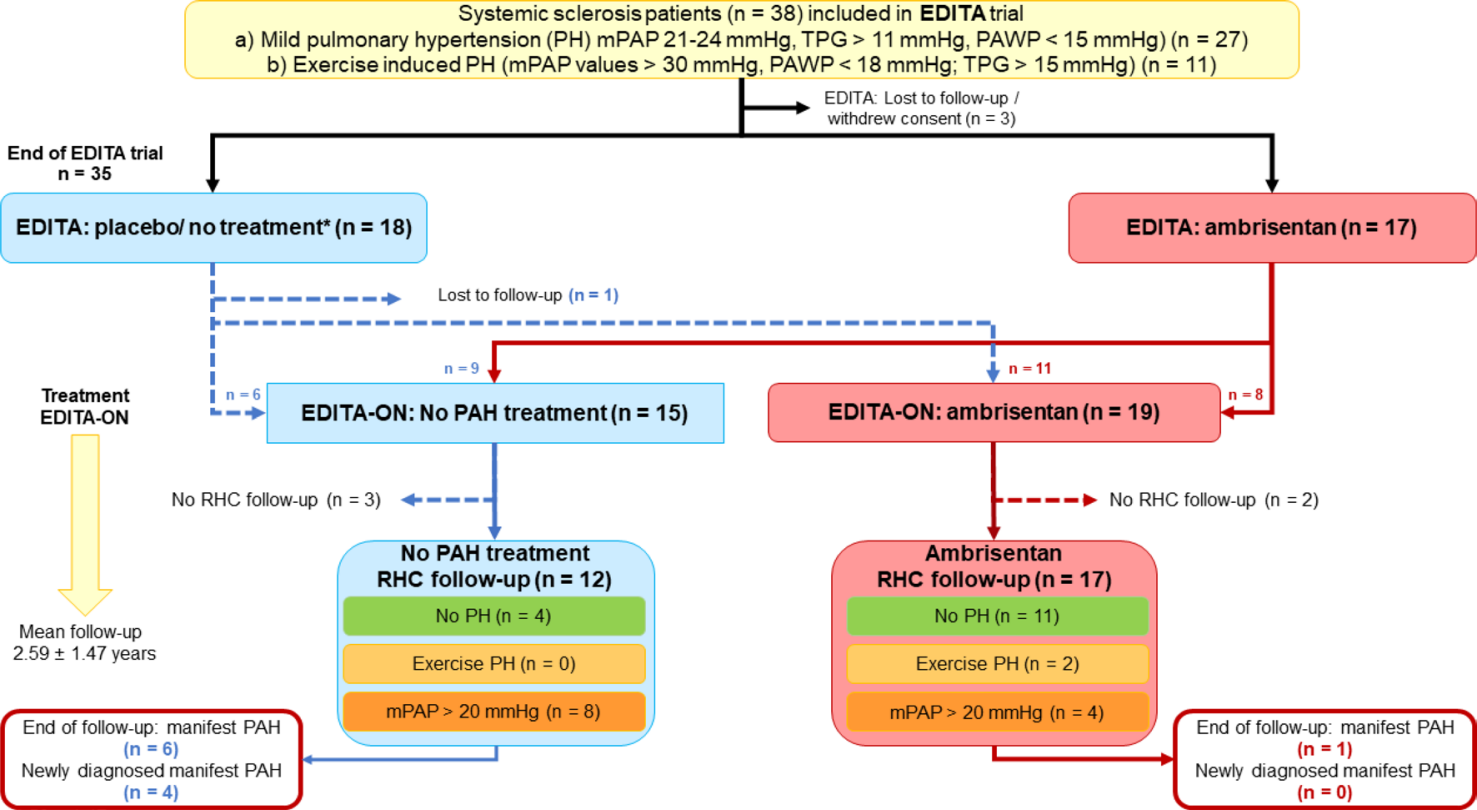
Study flow-chart. The graph gives information about the patient flow from baseline / end of the EDITA study until the last follow-up in the EDITA-ON study. Exercise PH: Δ (mPAP/cardiac output) > 3 mmHg/l/min, mPAP: mean pulmonary arterial pressure, PAH: pulmonary arterial hypertension, PAWP: pulmonary arterial wedge pressure, PH: pulmonary hypertension, RHC: right heart catheterization, TPG: transpulmonary gradient. *early termination patients from EDITA trial that continued follow-up (EDITA-ON). without treatment: from placebo group *n* = 2, from ambrisentan group *n* = 1

From 38 SSc patients participating in the EDITA study, three were lost to follow-up or withdrew consent before the end of the EDITA trial, while one further patient was lost to follow-up during long-term observation period (Fig. 1). Thus, a total of 34 SSc patients were included in the analysis of the EDITA-ON study (86.7% female, mean age 55 ± 11 years (Table 1), 81.8% WHO FC II and 14.3% WHO FC III, mean 6MWD 467.10 ± 83.99 m (m)). In total, 19 out of the 34 patients (55.8%) received ambrisentan open label during follow-up (EDITA-ON, Table 1; Fig. 1), eight continued treatment having had ambrisentan in the EDITA trial and 11, who were formerly in the placebo group, were switched to ambrisentan open label at the end of the EDITA trial (Fig. 1). Fifteen out of the 34 patients (44.1%) decided not to be treated with ambrisentan, nine after having received ambrisentan in the EDITA trial and six patients, who were formerly in the placebo group (Fig. 1). Treatment decisions were based on consensus between patient and physician. All patients who did not receive ambrisentan during follow-up wished not to receive treatment. No patients received vasodilative medication for treatment of Raynaud’s or digital ulcers.

There was no difference in the distribution or treatment of comorbidities (i.e. arterial hypertension, coronary heart disease) between the groups. (Table 1). In six patients diuretics were initiated during follow up (three under ambrisentan and three in the no PAH treatment group). During follow-up, in four patients left heart catheterisation was performed. Of these two (one ambrisentan, one no PAH treatment) had a stent.

The patients were followed-up for a period of 2.59 ± 1.47 years (Fig. 1). Overall, 29 patients underwent an evaluation with invasive hemodynamic measurements via RHC, 17 from the ambrisentan group, 12 from the control group after a median of 2.36 years, interquartile range = 1.81). Five patients did not wish any invasive evaluation during follow-up (Fig. 1). At baseline, mean mPAP was 19.8 ± 5.2 mmHg with a mean pulmonary arterial wedge pressure of 10.0 ± 3.8 mmHg and a mean pulmonary vascular resistance of 1.73 ± 0.72 WU (Table 1). Mean hemodynamics at baseline did not significantly differ between control and intervention group (Table 1).

### Change of clinical parameters (Table 2; Figs. 2 and 3)

**Table 2:**
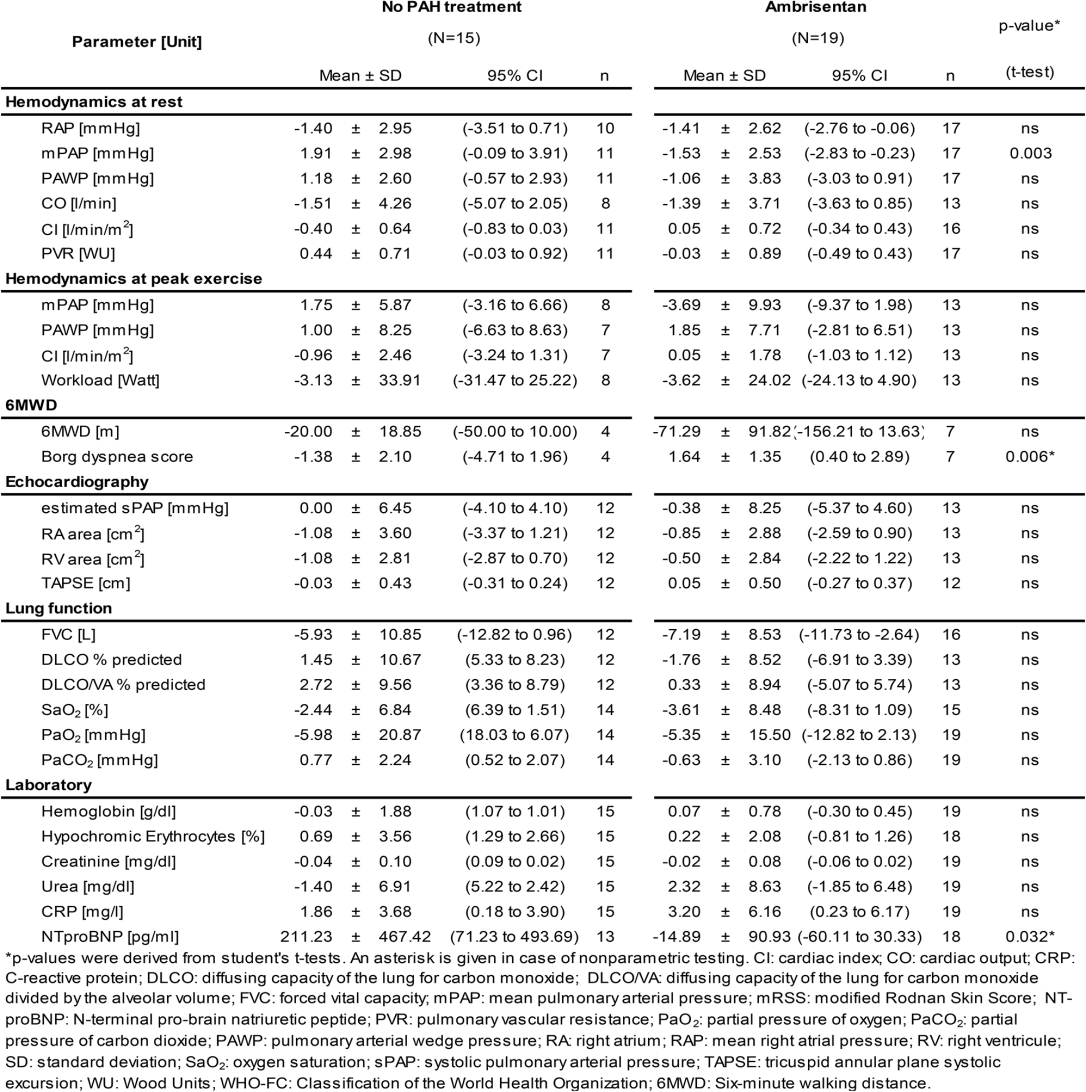
Changes in clinical parameters during follow-up

**Fig. 2:**
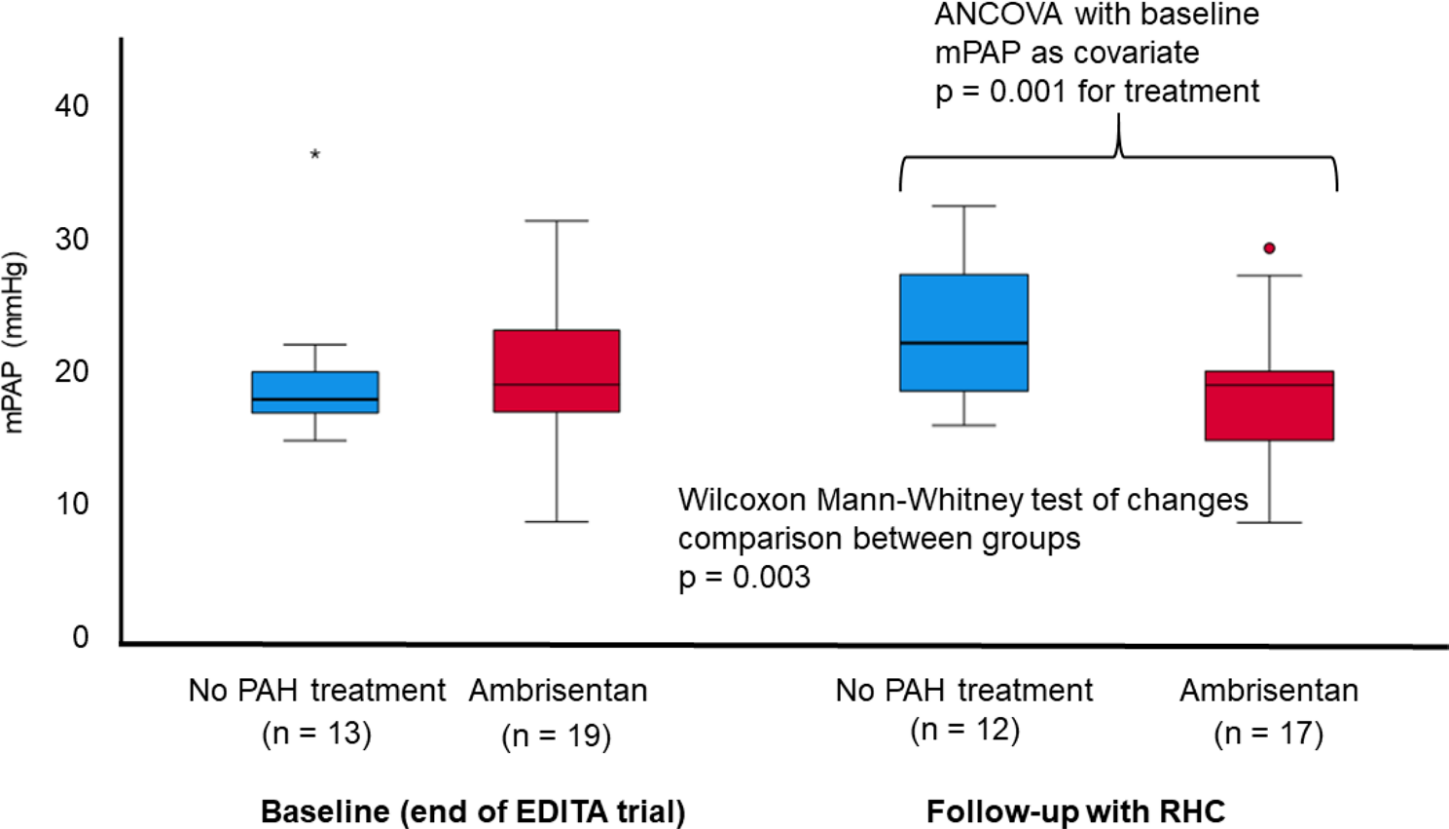
Change of mean pulmonary arterial pressure during long-term follow-up. Patients receiving ambrisentan significantly improved in their mPAP compared to patients who were treated without ambrisentan (*p* = 0.001)

**Fig. 3:**
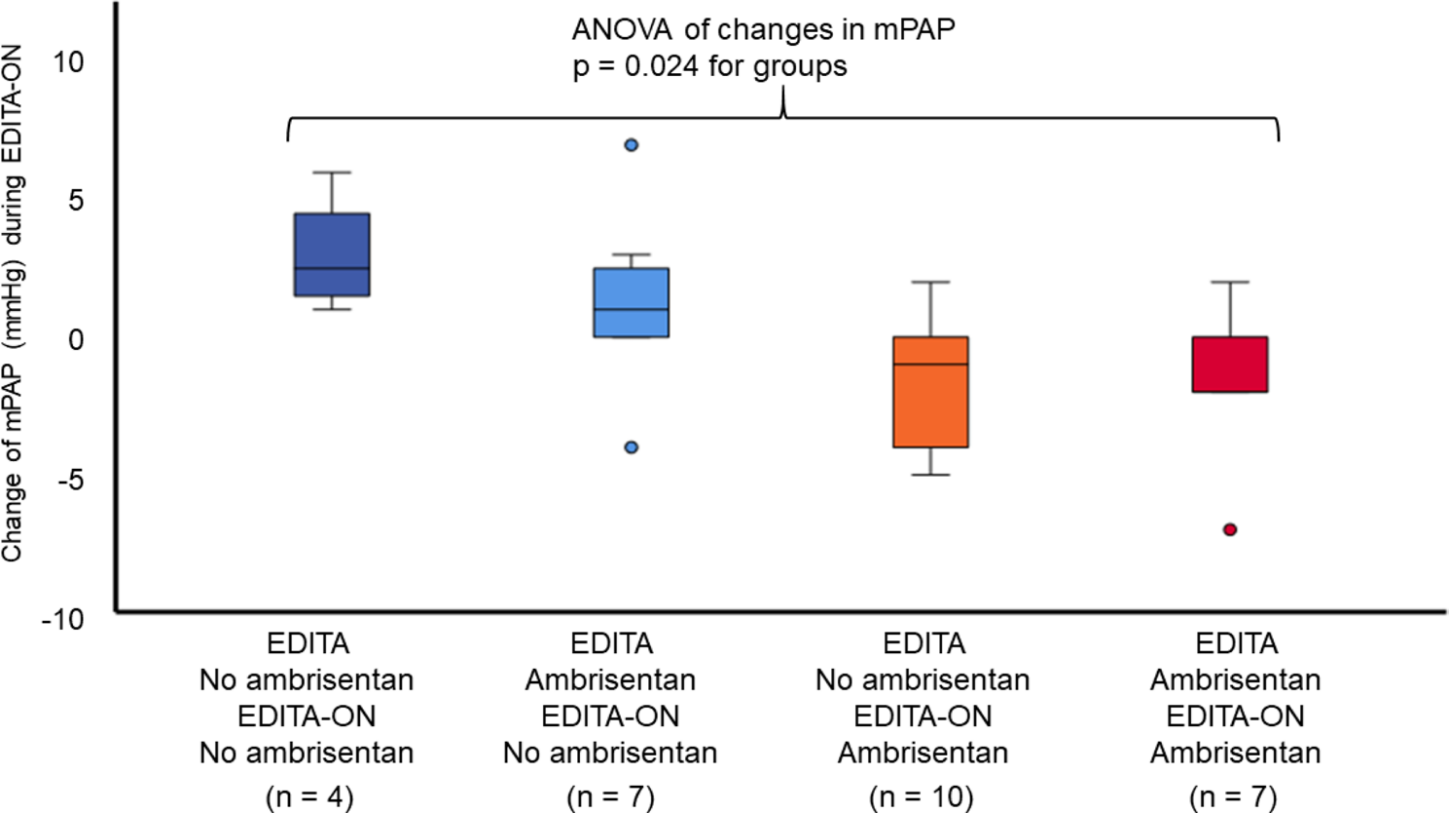
Change of mean pulmonary arterial pressure during long-term follow-up according to treatment subgroups. Patients receiving ambrisentan throughout EDITA-ON had a reduction in mPAP, while patients with no treatment in EDITA-ON showed an increase in mPAP (ANOVA *p* = 0.024)

During follow-up, patients receiving ambrisentan showed a statistically significant reduction in mPAP (-1.53 ± 2.53 mmHg) compared to patients of the control group (mPAP + 1.91 ± 2.98 mmHg, Fig. 2, *p* = 0.003). There was a reduction in mPAP for all patients who received ambrisentan during EDITA-ON (both patients with ambrisentan and no ambrisentan treatment during EDITA), but no reduction for patients who did not receive ambrisentan treatment during EDITA-ON (ANOVA *p* = 0.024). Changes of other hemodynamic parameters did not significantly differ between groups both at rest and during exercise (Table 2). Five of the patients in the ambrisentan group improved hemodynamically and had no signs of PVD at the end of EDITA-ON. In the control group none of the patients showed a comparable hemodynamic improvement.

Change of subjectively perceived exertion during 6MWD according to the Borg scale significantly differed between groups with an increase in the ambrisentan and a decrease in the control group (*p* = 0.006 Mann-Whitney-U-Test). Furthermore, patients in the ambrisentan group showed a significant decrease of NTproBNP compared to no PAH treatment (*p* = 0.032 Mann-Whitney-U-Test). Other clinical parameters did not significantly differ between groups.

### Outcome during follow-up (Table 3)

**Table 3:**
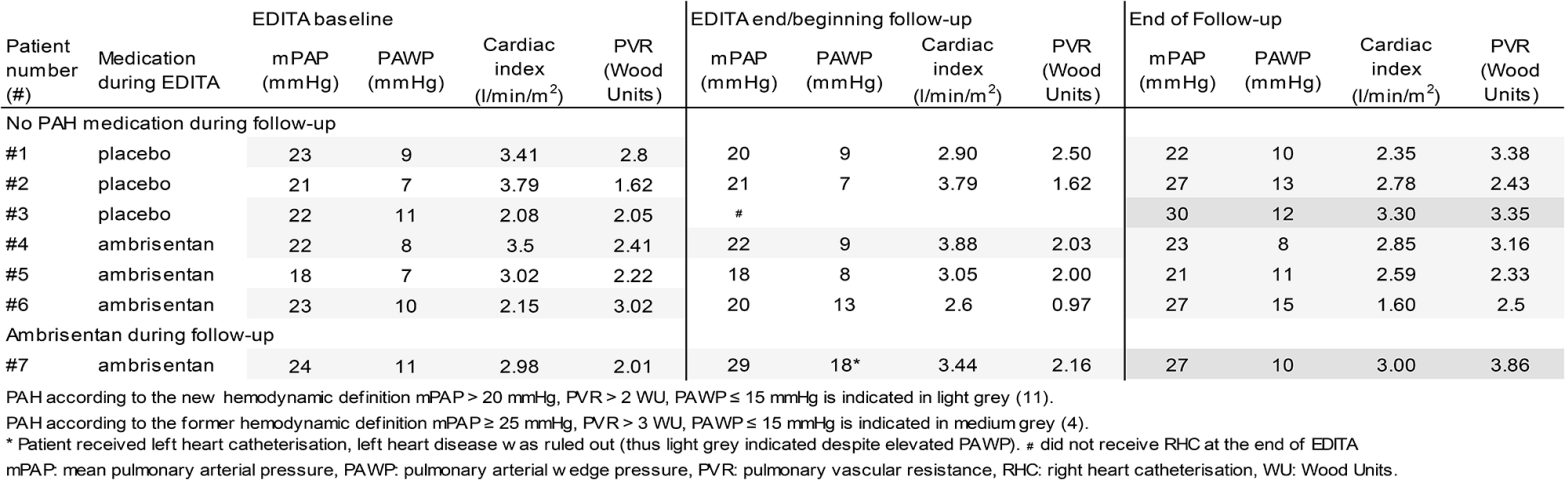
Individual development of hemodynamics in patients presenting with manifest PAH at the end of the observation period

At the end of follow-up, six (50%) of the 12 patients who did not receive PAH medication presented with manifest PAH according to the definition of the new European PH-guidelines, compared to 6% (one of 17) out of those patients who received ambrisentan during follow-up (*p* < 0.0001, Table 3; Fig. 1). Four of the 12 patients (33.3%) receiving no PAH treatment newly developed PAH during EDITA-ON follow-up vs. none receiving ambrisentan. One patient continuing ambrisentan since the beginning of the EDITA trial (patient #7, Table 3) showed mild worsening of mPAP and PVR and stable CI until the end of follow-up. The PAWP at the end of EDITA/baseline of EDITA-ON was elevated and normalized at the last follow-up. A left heart disease could be ruled out by left heart catheterization in the meanwhile.

Consequently, six out of seven patients presenting with manifest PAH at the end of the observation period did not receive ambrisentan during follow-up. Three were on placebo during EDITA (No 1–3, Table 3) and three were initially on ambrisentan (No 4–6, Table 3). The three patients never receiving PAH medication (placebo in EDITA) presented with worsening of hemodynamics from baseline of EDITA until their final follow-up. Three patients receiving ambrisentan during EDITA but not during follow-up showed worsening of hemodynamics after constant or even improved hemodynamics during ambrisentan treatment in EDITA (Table 3). In four patients receiving no PAH treatment, PAH developed denovo during follow-up, compared to none among patients receiving ambrisentan (*p* < 0.0001).

SSc patients receiving ambrisentan exhibited improvement in mPAP categories (mPAP < 21 mmHg, exercise-induced PH, mPAP > 20 mmHg) in four cases, while worsening of mPAP category was not detected within this group. The mPAP in the control group worsened in four patients and improved in none (*p* = 0.005). PVR improved in three patients ≤ 2 WU and worsened > 2 WU in two patients in the ambrisentan group, whereas in the control group there was no improvement and three patients worsened to values > 2 WU (*p* = 0.089).

### Safety and tolerability

Treatment with ambrisentan was well tolerated during EDITA-ON follow-up. Severe adverse events or treatment discontinuation did not occur in the intervention arm. Hemoglobin remained stable in the ambrisentan group (Table 2). Patients of the control group who developed PAH during follow-up received targeted PAH therapy. In the EDITA trial two patients from the ambrisentan group showed signs of left heart disease. One patient had a persistent isolated postcapillary PH at the end of EDITA-ON and one patient underwent an intervention for coronary heart disease (stent) and had a persistent PAH in the recent evaluation and was treated accordingly.

### Impact of the new definition

At the end of EDITA-ON, four patients were newly diagnosed with PAH according to the current guidelines [4], all from the group without therapy and received targeted PAH according to the guidelines.

Based on the former definition of PAH from 2016 [11], only one patient at baseline (end of EDITA trial) and two patients during follow-up (end of EDITA-ON) would have been diagnosed with PAH. Using the updated definition from 2022, six patients at baseline had PAH (data not shown) and seven presented with PAH at the end of long-term follow-up (Table 3).

## Discussion

This is the first study assessing the long-term treatment effect of therapy with ambrisentan in patients with mild pulmonary vascular disease. During long-term follow up none (out of 17) of the patients receiving ambrisentan newly developed a PAH in contrast to four (out of 12) patients without treatment (*p* < 0.0001). Furthermore, there was a significant deterioration of the mPAP in the control group (without ambrisentan) whereas none of the patients in the ambrisentan group worsened (*p* = 0.005). Five of the patients in the ambrisentan group even improved hemodynamics, showing no signs of PVD in the last follow-up. In the control group none of the patients showed a comparable improvement. It has to be noted that in this short interval patients without treatment developed already a severe PAH.

In the EDITA study a significant improvement of PVR but not mPAP was observed compared to placebo [9]. Furthermore, only patients from the placebo group developed an impairment of hemodynamics in need for targeted PAH therapy. During long-term follow-up, patients with PAH from the ambrisentan group exhibited improvement in RHC parameters whereas patients without PAH-medication deteriorated. Importantly, the discontinuation of the medication was followed by the development of PAH in three cases, according to the new definition. Thus, a discontinuation of PAH targeted therapy should be done with caution especially in high risk populations.

In a study assessing the effect of long term ambrisentan therapy in patients with PAH, a significant change of 6MWD and absence of clinical worsening or death were shown [12]. Hemodynamic evaluation of patients in long-term follow-up studies for ambrisentan (ARIES-1 and 2), though, was not performed [13]. In our study a significant change of 6MWD was not seen. This could be partly due to the mild disease, the impaired walking capability caused by arthralgia in some patients and the small study population. Clinical worsening of PH/PAH defined by hospitalisation was not reported by any of the patients, although worsening of hemodynamics is usually accompanied with clinical deterioration [14]. In this current study, SSc patients were diagnosed early and were treated at a stage prior to further deterioration of hemodynamics. Thus, an early treatment decision assisted and may have prevented the manifestation of PAH. The new 2022 definition of PAH [4] enables the classification and diagnosis of PVD at an earlier stage of the disease especially in this high-risk population. This was also shown in this study with an impressive difference in the number of new diagnoses (one according to the former definition compared with five according to the new definition at baseline and two versus seven at follow-up), accordingly, as shown in previous studies [15–18]. Early detection of the disease may therefore enable early treatment and prevention of clinical and hemodynamic worsening.

### Limitations

The main limitation of this study involves its retrospective, open label, and single centre nature, with a small number of patients. Thus, a generalization of the results cannot be made. Furthermore, the patients were not blinded during follow-up, as the medication was prescribed. This might lead to biased results. As treatment decisions were discussed openly between the physician and the patient, a selection bias cannot be excluded. However, treatment decisions were derived in consensus between patient and physician and all patients who did not receive open label ambrisentan during follow-up wished not to receive treatment.

Patients of the ambrisentan group had significantly higher DLCO/VA at baseline, which may have influenced the results, as this is a risk factor for the development of PAH. Larger and multicentre studies with a longer observation period are needed. The efficacy and safety of riociguat in incipient pulmonary vascular disease are currently being tested in the double-blind, randomized, multicentre, multinational, placebo-controlled phase IIa ESRA study (NCT05339087).

The main strengths of this study are the low number of patients lost during follow-up. Overall, 34 of 35 patients continued follow-up at an expert PH center in the study and 29 of them received a RHC within the follow-up period.

In general, connective tissue disease patients, especially patients with SSc are at high risk for development of PAH. Early diagnosis and early treatment based on the current definition of PAH may prevent further deterioration and additional clinical worsening. PVD may develop at any stage of SSc [2], thus regular screening is required. The current guidelines suggest thorough evaluation of connective tissue disease patients with SSc characteristics including RHC in case of any doubt [4]. In this study patients with a low DETECT score [3] were assessed due to symptoms by RHC and could be treated with targeted medication. Thus, an interdisciplinary approach including rheumatologists and PH experts should be performed in this high-risk group.

## Conclusion

In this SSc cohort, continued therapy with ambrisentan significantly reduced worsening of hemodynamics compared to patients receiving no PAH treatment, indicating that early treatment and close follow-up could be beneficial in this risk group for developing PAH. Future trials are needed to confirm these results.

## Data Availability

The datasets used and/or analyses from the current study are available from the corresponding author on reasonable request.

## Abbreviations

CI: Cardiac index
CO: Cardiac output
CRP: C-reactive protein
DLCO: Diffusion capacity of carbon monoxide
DLCO VA: Diffusing capacity of the lung for carbon monoxide divided by the alveolar volume
dPAP: Diastolic pulmonary arterial pressure
FC: Functional class
FVC: Forced vital capacity
mPAP: Mean pulmonary arterial pressure
mRSS: Modified Rodnan Skin Score
NTproBNP: N-terminal pro brain natriuretic peptide levels
PaO_2_: Partial pressure of oxygen
PaCO_2_: Partial pressure of carbon dioxide
PAH: Pulmonary arterial hypertension
PAWP: Pulmonary arterial wedge pressure
PH: Pulmonary hypertension
PVD: Pulmonary vascular disease
PVR: Pulmonary vascular resistance
RA: Right atrium
RAP: Right atrial pressure
RHC: Right heart catheterization
RV: Right ventricle
SaO_2_: Oxygen saturation
SD: Standard deviation
sPAP: Systolic pulmonary arterial pressure
SSc: Systemic sclerosis
TAPSE: Tricuspid annular plane systolic excursion
WHO: World Health Organization
WU: Wood Units
6MWD: 6-minute walking distance
Δ (Delta): Difference
